# Supplementary material for: The initial development and validation of the Social Adaptability Skills Questionnaire: SASQ
Source: PLoS One. 2023 Aug 18;18(8):e0281971. doi: 10.1371/journal.pone.0281971 (PMC10437787; doi:10.1371/journal.pone.0281971)
Supplement: S1 Appendix — (DOCX) [file pone.0281971.s002.docx]

**Appendix A (SASQ 17-Item Questionnaire)**

**Q1**.  Je trouve que les relations entre les membres d’une équipe ne sont jamais spontanées mais toujours intéressées*. I find that relations between teammates are not usually spontaneous but always interesting.**»*

**Q2**. Lorsque l'entraîneur(e) me donne des ordres/conseils, je suis capable de suivre ses indications sans difficulté*. Whenever the coach gives me orders/advice, I am able to listen and respect his/her decision without difficulties.*

**Q3**.  J’ai du mal à écouter les entraîneurs(es) que je ne connais pas bien. *I have difficulties listening to coaches/trainers that I don’t know.*

**Q4**. Cela ne me dérangerait pas de me faire coacher dans une langue étrangère*. I wouldn’t mind being coached in a foreign language.*

**Q5**. Lorsque j'ai des coéquipiers et coéquipières d'origines ethnique et culturelles différentes, je me sens à l'aise pour communiquer avec eux. *Whenever I have teammates from a different cultural background, I feel at ease communicating with them.*

**Q6**. Je n’aime pas qu'il y ait des joueurs étrangers dans mon équipe *« I don’t like having foreign teammates**.*

**Q7**. Je ne supporte pas qu’un entraîneur(e) favorise certain-e-s joueur.se.s au détriment d’autres*. « I can’t tolerate it when trainer/coach favours certain players more than others.*

**Q8**. Je trouve normal que les temps de jeu en match puissent varier quand les entraîneurs (es) changent*. I find it normal that play time can vary whenever trainers/coaches change.*

**Q9**. Je suis paralysé lorsque mon entraîneur(e) me met la pression pour atteindre des objectifs. *I always feel paralysed whenever my trainer/coach puts pressure on me to reach my sporting goals.*

**Q10**. Je suis stimulé lorsque mon entraîneur(e) me met la pression pour atteindre des objectifs. *I always feel stimulated whenever my trainer/coach puts pressure on me to reach my sporting goal**s.*

**Q11**. Je me sens capable de m’entraîner avec n’importe quel entraîneur quels que soient ses comportements (pression, soutien, indifférence). *I feel capable of carrying out my training despite the coach’s behaviour (pressure, support, or indifference).*

**Q12**. Je suis toujours motivé-e à pratiquer mon sport, même si je trouve que l’entrainement proposé par l’entraîneur (e) n’est pas de bonne qualité. *I* *am always motivated to practice my sport even when the trainings proposed by the trainer/coach are not of high quality**.*

**Q13**. Je me sens vite perdu lorsque je suis loin de ma famille (exemple : pendant des stages ou camps loin de chez moi*).* *I always feel lost when I am away from my family* *(for example, during internships or camps away from my family.*

**Q14**. Je n’éprouve pas de difficultés particulières lorsque je suis loin de ma famille (exemple : pendant des stages ou camps loin de chez moi). *I don’t encounter any particular difficulties whenever I am away from my family (for example, during internships or camps away from home.*

**Q15**. Ce n’est pas envisageable pour moi que de vivre dans le futur loin de ma famille*. I don’t see myself living far from my family* *in the future.*

**Q16**.  Je n’accepterais pas de jouer dans un club qui n’a pas d’installations sportives de qualité*. I wouldn’t accept to play in a club whose training facilities are not of high quality.*

**Q17**. Je sais m’entraîner sérieusement même si la qualité des installations sportives n’est pas toujours satisfaisante. *I know how to train seriously on my own even when the sporting facilities are not satisfactory.*
