# Supplementary material for: The initial development and validation of the Social Adaptability Skills Questionnaire: SASQ
Source: PLoS One. 2023 Aug 18;18(8):e0281971. doi: 10.1371/journal.pone.0281971 (PMC10437787; doi:10.1371/journal.pone.0281971)
Supplement: Attachment — Submitted filename: Response to Reviewers.docx [file pone.0281971.s005.docx]

**Response to editor/s comments :**

| *Comment* | *Response* |
| --- | --- |
| 1.Ensure manuscript meets The PLOS ONE’S style (file naming) | File naming has been adjusted |
| 2.Amend the ethics statement to address:  (a) if participants provided written or verbal consent to participate in the study? | 2 (a) -Yes, this has been amended in each study part: (*After receiving approval from the* *Research Ethics Commission of the University of Lausanne (Project number: E_SSP_122021_00002) and informed written consent from all participants*). |
| 3. Provide minimal data set underlying the results. | We have provided minimal data set under file name “*Supporting Information Files*” (i.e., S1 Raw Dataset, S2 T-score Dataset, S3 Test-retest dataset) |
| 4. Amend the title on the online submission form to correspond the manuscript | This has been amended so that both the online and manuscript title correspond  (*The Initial Development and Validation of the Social Adaptability Skills Questionnaire: SASQ)* |
| 5. Include captions for the supporting information files at the end of the manuscript. | The following captions have been included at the end of the manuscript; (S1 Raw Dataset, S2 T-score Dataset, S3 Test-retest dataset) |

**Response to Reviewer 1 comments:**

| *Comment* | *Response* |
| --- | --- |
| *Line 58*: cite more than one study since you wrote “studies” | The sentence has been rephrased to include only one reference (*and a recent study has underlined distinct periods of adaptation to the new environment (2*). |
| *Line 61*: such as? Clarify sentence and add citation. | The sentence has been clarified as follows and citation added too:  (*as they may lack some of the basic skills and psychosocial resources (example: positive thinking, goal setting skills, self-confidence skills) that are needed to ensure successful moves) (2)* |
| *Line 78*: Indicate citation | Citation has been included (*Hauw et al., 2022*) (6). |
| *Line 85*: Indicate citation | Citation has been included (*Abbot & Collins, 2004 (7); Toering et al., 2011 (8); Van Yperen, 2009 (9*). |
| *Line 222:* Why did the authors change the Likert-type scale? | We do acknowledge that in Lei Chang (1994) while evaluating which point scale between 4 or 6 in relation to reliability and validity argued for the use of a 4 point scale. However, the authors do admit as a limitation in their study that respondent knowledge with respect to what is being measured might have affected their conclusion as to whether the 4 point scale or 6 point scale was the best choice.  Additionally, the authors do encourage future studies to look beyond a simple relation between number of scale points and reliability or validity for possible interaction effects between scale points and other factors such as respondent knowledge of what is being measured .  Confronted with this limitation, we chose to apply the 6 point scale which had previously been applied in similar context as the current study (Development & Initial validation of the psychological characteristics of developing questionnaire-PCDEQ (MacNamara & Collins, 2011). |
| *Line 249*: Did the authors conduct a priori sample calculations? | Yes we did and we have now inserted this part in the manuscript.  The number of subjects needed to perform the EFA. The 10:1 ratio (number of subjects for each item in the questionnaire) is the recommended number and is the one that generates some, yet not full consensus in the literature (DeVellis, 2003; Hill and Hill, 2005; Henson and Roberts, 2006; Kahn, 2006; Worthington and Whittaker, 2006; Hair et al., 2019). However, there are also authors who mention minimum ratios of 5:1 (Henson and Roberts, 2006; Hair et al., 2019) and others that mention absolute values: 50 very poor; 100 poor; 200 acceptable; 300 good (Tabachnick and Fidell, 2019).  We followed the recommendations of mostly used in the literature (person per item ratio – 10:1)  *In general, there is some large agreement that larger samples are likely to result in more stable correlations among variables and will result in greater replicability of EFA outcomes. Therefore since SASQ contained 34 items, the current study required between 340- 510 participants which fulfilled the empirical rule.* |
| How was the data collected? Online questionnaire or paper and pencil style. | The data was collected using paper-and-pencil style.  The manuscript has been amended to show this (*Self-administered paper-pencil SASQ questionnaires were distributed to the 543 participants at their respective clubs between March and May 2022*). |
| Table 2: Report Skewness and Kurtosis and missing values for each item for transparency. | Table 2 has been amended to include both Skewness and Kurtosis and missing values. |
| At this point, the reviewer is unable to know which items refer to which factor (Table 3) | Each of the four factors have been named and corresponding item question inserted: (Coach, Teammates, Family/friends, and Club). |
| How many factors did EFA extract? disclose | The EFA extracted 4 factors. We have provided a *scree plot* and a total *variance explained* table at the end of this document. |
| Table 3: There are several cross-loadings, how did the authors handle such cases? | We took two useful steps of looking for (a) correlations that were not high enough, and (ii) correlations that were high.  *Low correlations*- We visually scanned the correlation matrix and looked for correlations below 0.3, which normally should be excluded. However, this approach isn’t helpful since small correlations will be significant in large samples like ours. This approach is subjective, but since data analysis is a skill, we focused more on objective approach by testing whether the correlation matrix resembled the identity matrix (this was done by visually checking the diagonal components and they were reported to be zero.  *High correlations*- High cross-loadings could be due to high multicollinearity, we therefore checked for multicollinearity by looking at the determinant of *R*-matrix. The determinant of the *R*-matrix should be greater than 0.00001 (Field, 2018). Our determinant was .001. In any case multicollinearity does not cause a problem for principal component analysis but was verified to help arrive at informed decision.  We were also guided by the fact that SASQ questionnaire measure the same underlying dimension of “*social adaptability skills*” and we expected the dimensions to correlate with each other since the sub-components measure the same thing. We will provide two examples to support our argument: (example 1: *adapting to coach, teammates, club, or being away from family and friends all measure a person’s overall social adaptability skill*. Example 2: *when measuring a person’s overall anxiety in terms of sub-components such as worry, intrusive thoughts, and physiological arousal*), there should still be moderate correlations between the variables relating to these sub-traits. Therefore, we wouldn’t expect to see variables that have small correlations with each other. |
| *Line 331*: Alpha for which factors? General? This needs to be clarified. | This has been clarified as the general alpha and included in the manuscript as follows: (*The general reliability static was α = 0.876, indicating good reliability (38, 39*). |
| *Line 347-350:* This section lacks significant information regarding CFA. Report the fit indexes and cut-offs, disclose estimator selected, and report sample size calculations. | We have amended the section to answer the reviewers request as follows:  The analysis was conducted using AMOS version 27. The CFA is presented in route diagrams where the circles represent latent variables and the squares represent the observed variables (38, 39, 40). Two-headed arrows indicate covariance between the four latent variables, and single-headed arrows indicate the assumed direction of influence (41). Within the literature, the most used approximation indices include (Hoyle and Panter, 1995; Worthington and Whitaker, 2006; Brown, 2015) (a) the *absolute indices* referring to the degree to which the covariances implied in the model match the observed covariances through which the free parameters are estimated, and (b) *incremental indices* which refers to the degree to which the model is superior to the alternative model, usually where no covariances between variables are specified- null or independent model.  Despite reporting both the absolute and incremental indices in the current study, the CFA model was validated using the absolute indices (i.e., normalized Chi-Square- X^2^/df and Root Mean Squared Error of Approximation-RMSEA) since an optimal fit is indicated by values close to zero. Normalized Chi-Square (X^2^) values < 3.0 indicate reasonable adjustment (Arbuckle, 2013; Hair et al., 2019) with a value of 5.0 being the minimum acceptable (Bentler, 2002). As concerns RMSEA, values of ≤ 0.06 indicate an adequacy of the model (Hu and Bantler, 1999), but normally the most used cut-off values are (Brown, 2015; Bryne, 2016; Kline, 2016): ≤ 0.05 good fit, ≤ 0.08 acceptable fit, ≤ 0.10 indicate a mediocre fit, and > 0.10 a poor (un acceptable fit). Factor weights (FL; factor loading) were also computed with values equal to or greater (≥ 0.5) being accepted (26). Ideally, according to Hair et al., 2019) accepted values should be over 0.7.  *Sample Size Calculation:*  We followed the recommendations by DeVellis, 2003 of participant item ratio of 10:1 in estimating the sample size. |
| *Line 360-365:* Several issues to be checked regarding model fit indexes (recommendations by Hu & Bentler, 1999).  Include modern approaches of model fit indices (Peugh & Feldon, 2020) | This part has been addressed in the preceding response (part of line 347-350 response).  Thanks for the suggestion, updates have been made. |
| *Figure 1:* Several problems:  (a). correlating measurement errors not acceptable as it forces the model to fit.  (b). covariances are above 1  (c) several low factor loadings are reported  (d). two items are insufficient to provide enough reliability and validity of the factor (Hair et al., 2019) | Thanks for your suggestion, we have adjusted the model (Figure 1) by deleting the correlating measurement errors and re-run the *AMOS* analysis.  This has been corrected by reporting standardized loadings. This occurred due to an error on our side by reporting unstandardized values (Thanks once again for the observation).  This occurred due to our reporting of the unstandardized values; It has since been corrected as follows (*Last, the latent variables for the four subscales of the SAS had nine, three, three, and two items each (Figure 1). According to the route diagram, all items show acceptable factor loads (standardized values) ranging from 0.66 to 2.74, except for teammates factor (Q12R) which had a value of 0.31. Nine elements of the coach construct had factorial loads between 1.00 and 2.74, as did the three elements of the teammates construct, which had factorial loads between 0.31 and 1.00. The three elements of the family construct ranged from 0.95 to 1.13, and the last two elements of the club construct ranged from 0.66 to 1.00).*  We do agree with your comment, however, we provide the following statement to support our decision:  *It should be noted that good practice dictates a minimum of three items per factor (Raubenheimer, (2004).). Three of the SASQ factors consisted of at least three items per factor, although the club factor had only two items. In retaining the club factor with only two items, we present the following arguments: First, there was a strong theoretical and practical reason linked to the observation that future athletes are already self-motivated to pursue their careers despite problematic challenges concerning the club. Second, our decision is supported by previous research in the psychology literature suggesting that constructs that do not have a wide domain or those that are not conceptualized as multidimensional may present single-item measures (Bergkvist & Rossiter, (2007);  Drolet, & Morrison, (2001); Gosling et al., 2003; Winous et al., 1997). Third, a factor with two items is only considered reliable when the items are highly correlated (r = 0.70) but fairly uncorrelated with the other items, as was the case in the current study (Young & Pearce, 2013).* |

According to the scree plot, it is a little difficult to interpret because there are points of inflexion at both 5 and 8 factors, meaning we could justify retaining either between 4 or 5 factors. The recommendations for Kaiser’s criterion are for much smaller samples than we have. Given our huge sample and that there is some consistency between Kaiser’s criterion and the scree plot, it is reasonable to extract 4 factors.

**Table 5.** Grouping of the 34 items into four factors with rotated factor loads

| **Total Variance Explained** | | | | | | |
| --- | --- | --- | --- | --- | --- | --- |
| Component | Initial Eigenvalues | | | Extraction Sums of Squared Loadings | | |
|  | Total | % of Variance | Cumulative % | Total | % of Variance | Cumulative % |
| 1 | 7.688 | 22.611 | 22.611 | 7.688 | 22.611 | 22.611 |
| 2 | 2.310 | 6.795 | 29.406 | 2.310 | 6.795 | 29.406 |
| 3 | 1.762 | 5.183 | 34.589 | 1.762 | 5.183 | 34.589 |
| 4 | 1.360 | 3.999 | 38.588 | 1.360 | 3.999 | 38.588 |
| 5 | 1.235 | 3.631 | 42.219 |  |  |  |
| 6 | 1.201 | 3.532 | 45.751 |  |  |  |
| 7 | 1.116 | 3.282 | 49.034 |  |  |  |
| 8 | 1.041 | 3.061 | 52.094 |  |  |  |
| 9 | .963 | 2.833 | 54.927 |  |  |  |
| 10 | .915 | 2.693 | 57.620 |  |  |  |
| 11 | .879 | 2.585 | 60.204 |  |  |  |
| 12 | .859 | 2.526 | 62.730 |  |  |  |
| 13 | .838 | 2.466 | 65.196 |  |  |  |
| 14 | .822 | 2.416 | 67.612 |  |  |  |
| 15 | .762 | 2.241 | 69.854 |  |  |  |
| 16 | .731 | 2.150 | 72.004 |  |  |  |
| 17 | .707 | 2.081 | 74.084 |  |  |  |
| 18 | .668 | 1.965 | 76.049 |  |  |  |
| 19 | .654 | 1.924 | 77.973 |  |  |  |
| 20 | .638 | 1.878 | 79.851 |  |  |  |
| 21 | .633 | 1.863 | 81.714 |  |  |  |
| 22 | .618 | 1.818 | 83.531 |  |  |  |
| 23 | .603 | 1.773 | 85.304 |  |  |  |
| 24 | .580 | 1.706 | 87.010 |  |  |  |
| 25 | .546 | 1.607 | 88.617 |  |  |  |
| 26 | .511 | 1.503 | 90.121 |  |  |  |
| 27 | .503 | 1.480 | 91.600 |  |  |  |
| 28 | .478 | 1.407 | 93.007 |  |  |  |
| 29 | .451 | 1.327 | 94.334 |  |  |  |
| 30 | .443 | 1.303 | 95.638 |  |  |  |
| 31 | .429 | 1.262 | 96.899 |  |  |  |
| 32 | .398 | 1.170 | 98.069 |  |  |  |
| 33 | .357 | 1.051 | 99.120 |  |  |  |
| 34 | .299 | .880 | 100.000 |  |  |  |
| Extraction Method: Principal Component Analysis. | | | | | | |

The eigenvalues associated with each factor represented the variance explained by that particular factor. After translating the eigenvalues into percentage, factor 1 explained relatively large amount 22.611% of the total variance, whereas subsequent factors explained only small amounts of the total variance: *factor 2*-6.795%, *factor 3*- 5.183%, and *factor 4*- 3.999.
