# Supplementary material for: The initial development and validation of the Social Adaptability Skills Questionnaire: SASQ
Source: PLoS One. 2023 Aug 18;18(8):e0281971. doi: 10.1371/journal.pone.0281971 (PMC10437787; doi:10.1371/journal.pone.0281971)
Supplement: Attachment — Submitted filename: Rebuttal Letter-Response to Reviewers.docx [file pone.0281971.s006.docx]

**Rebuttal Letter (Response to reviewers).**

First, we would like to thank both reviewer 1 & 2 for their insightful comments.

| **Question:** | **Response** |
| --- | --- |
| Q1. Make major changes by either:  -using formative analysis (PLS-SEM) or  -creating some sort of inventory or index to indicate extent of athletes adaptation.  Choice of whether to apply reflexive or transformative analysis- PLS-SEM? | In light of Reviewer 2 proposal, and having previously confirmed the SASQ factors through CFA, we further performed a predictive validity analysis on the data using Latent Class Analysis (LCA). Our justification behind applying the LCA was to complement the 17-item SASQ factorial structure previously established. In addition, LCA identified three classes of SAS (low achievers, average achievers, and high achievers) with four discriminating dimensions (i.e., coach, teammates, family, and club) which could be used to indicate the extent of athletes adaptation. The combination of the two methodologies constitute an exploratory sequential mixed methodology design approach to integrate social adaptability skills during athletes change from club to club. Similar mixed methodology has been previously applied with success in determining the internal and predictive validity on psychometric instrument (The Health of the Nation Outcome Scale- HoNOS) (Golay, Basterrechea et al., 2016).  In addition, the current study settled on LCA because it transcends other conventional parametric analyses (i.e., formative PLS-SEM) by relaxing the assumptions of homogeneity of variance, skewness, normal distribution, and linearity that cannot be analysed using the same conventional parametric clustering techniques (Magidson & Vermunt, 2016). The analysis was performed on Latent Gold® 6.0 (Magidson & Vermunt, 2016) with the maximum likelihood method for parameter estimation.  The LCA in the present study comprised: (a) building and classification of a cluster model (b) bootstrapping to confirm optimum fit model, and (c) predicting the precision of the optimal model using classification statistics. The optimal model was determined by comparing the fit statistics of three models (Fraley and Raftery, 1998) namely (AIC, CAIC, and BIC).  *Results*  Four models were fitted to the data, a three class model differentiating between three different groups (low achievers, average achievers, and high achievers) had an optimal fit to the data (BIC = 4172.964; AIC = 3437.369; CAIC = 3642.426). The bootstrapping *p* value indicated good fit hence confirming results of CFA.  Concerns have been raised about considering psychometric measures as non-interdependent scales because the reliability of single-item scales can be legitimately questioned ( Speak, Hay et al., 2015). This suggestion extends to the SASQ-17 items which cannot be considered as uncorrelated (Muncer & Speak). However, since validity implies reliability (Furr & Bacharach, 2014; Gavin, 2008) the choice of the most relevant SASQ items were based on pragmatic grounds including their predictive value for relevant outcomes. It is therefore essential to emphasize the importance of external validity when choosing the most suitable SASQ scores, some of which should be correlated or not with each other. Adequate SASQ scores should not be selected to internal validity evidence alone reason why it was deemed appropriate to apply CFA and complimented by LCA in the current study.  It should be noted that we applied the formative PLS on our data and the results were not quite convincing (low values). Low values could have been due to residual variance. Therefore, it was deemed justified to apply the LCA instead of formative PLS. |
| Q2. Justification of unbalanced factors between 2-9 items per factor? | During coding, four major themes (i.e., *coach*, *teammates, family, and coach*) emerged. Each major theme had several sub-themes (i.e., *coach with 8 sub-themes, teammates with 3 sub-themes, family with 4 sub-themes, and club 3 sub-themes*). From this presentation, it can be seen that the coach as a major theme with 8 sub-themes generated more questions as compared to the other three major themes (leading to more items on the coach factor). An excerpt from study one below provides a detailed information of each major theme and its corresponding sub-themes.  These SAS structures were organized into four factors (i.e., coach, teammates, family/friends, and club). The sub-themes in relation to the coach were: *obeying orders, language barrier, inequality/favouritism, reduced play time, difficulty combining sports and other life domains, pressure to perform, lack of structured training,* and *low-level competition*. Sub-themes related to teammates included: *respect, language barrier,* and *negative perception of others*. This was followed by sub-themes related to family/friends, which included: *climate change, distance, language* and *culture shock barrier*. Last, club included the following sub-themes: *poor facilities and logistics* and *lack of support*. |
| Q3: Concerns about study 1 sample size of 20 participants only). | First, we stopped collecting data once we reached data saturation point with no new items emerging from the themes. Our choice is supported by the following statement: *In accordance with the criterion of data saturation (Corbin & Strauss, 2015; Holt & Tamminen, 2010; Strauss & Corbin, 1998; Weed, 2009) data collection and analysis were discontinued when the categories upon which the theory was built no longer producing new insights*.  Second, participants in study 1 were selected through purposive sampling to help achieve the study goals. *For example, in order to build the family theme where elite athletes encountered difficulties of being away from their family and friends, we had to recruit elite athletes who had come from overseas to play in their adopted country*. For this reason, “few” participants as many as 20 were deemed enough once the data saturation point was attained. |
| Q4: Why was no attention given to exorbitant skewness on items 11 and 12? | Thanks for the insight,:  This was an error off omission from our part to indicate the treatment performed.  The treatment involved-  An appropriate transformations using a Box-cox normality plot was applied to find the transformation that approximately normalised the data. This was further verified by computing the correlation coefficient of a normal probability plot.  The following sentence has been added on the manuscript to indicate the transformation applied-  (*Items 11 and 12 did not follow the normal distribution and as such, a Box-cox normality plot was applied to find a transformation that approximately normalised the data. This process was then verified by computing the correlation coefficient of a normal probability plot*) |
| Q5: *Reviewers concern as regards the positive and negatively worded items on SASQ* | The initial SASQ 34-item list contained a mixture of negatively (*N* = 13) and positively (*N* = 21) worded questions to minimize the danger of acquiescence bias. The phenomenon of acquiescence bias is well known since it represents participants preference to always rate on the positive side regardless of item content (Weijters et al., 2013). This was the reason why SASQ presented a mixture of both positively and negatively worded questions. In addition, some negatively worded items were confirmed in CFA. |
| **Minor changes**  Q6: Item justification- Expert panel. Were all items scoring below 4 deleted? | We take note of your comment and thank you in advance.  The sentence has been corrected so that it does not give the misconception that all items that scored below 4 were deleted, it reads as follows: *A second expert panel was constituted and asked to follow the same procedure, and this resulted in the rewording of several items (N = 25) and deletion of several others (N =14).* |
| Q7: (five additional items were added). How were these developed? | The interviews were reviewed once again resulting into five additional items being added).  The paragraph has been adjusted as follows:  Items that were rated 4: *relevant* or less were discussed by the whole panel (4). At the end of this stage, some of these items were marked for deletion. A first panel review resulted in the rewording of several items (*N* = 41) due to grammatical errors and comprehension issues. A total of ten items were deleted and five additional items were added. Furthermore, the five items that were added were part of the interviews and not from the expert panel. A second expert panel was constituted and asked to follow the same procedure, and this resulted in the rewording of several items (*N* = 25) and deletion of several others (*N* =14). As a result of this stage, 36 items were included in the SASQ. |

**References:**

Corbin, J. M., & Strauss, A. L. (2015). *Basics of qualitative research: Techniques and procedures for developing grounded theory* (4th ed.). Los Angeles: SAGE.

Fraley, C. (1998). How many clusters? Which clustering method? Answers via model-based cluster analysis. *The Computer Journal*, *41*(8), 578–588. <https://doi.org/10.1093/comjnl/41.8.578>.

Furr, R. M., & Bacharach, V. R. (2014). *Psychometrics: An introduction* (Second edition). SAGE.

Gavin, H. (2008). *Understanding research methods and statistics in psychology*. SAGE Publications.

Golay, P., Basterrechea, L., Conus, P., & Bonsack, C. (2016). Internal and predictive validity of the French health of the nation outcome scales: Need for future directions. *PLOS ONE*, *11*(8), e0160360. <https://doi.org/10.1371/journal.pone.0160360>

Holt, N. L., & Tamminen, K. A. (2010). Moving forward with grounded theory in sport and exercise psychology. *Psychology of Sport & Exercise, 11*(6), 419-422. doi:10.1016/j.psychsport.2010.07.009.

Strauss, A., & Corbin, J. (1998). *Basics of qualitative research techniques*. Thousand Oaks, CA: Sage publications.

Magidson, J., & Vermunt, J. K. (2016). *Latent Gold [Computer software]*. Belmont, MA: Statistical Innovations.

Speak, B. L., Hay, P., & Muncer, S. J. (2015). HoNOS – their utility for payment by results in mental health. *International Journal of Health Care Quality Assurance*, *28*(2), 115–128. <https://doi.org/10.1108/IJHCQA-08-2013-0096>.

Weed, M. (2009). Research quality considerations for grounded theory research in sport & exercise psychology. *Psychology of Sport & Exercise, 10*(5), 502-510. doi:10.1016/j.psychsport.2009.02.007.

Weijters, B., Baumgartner, H., and Schillewaert, N. (2013). Reversed item bias: an integrative model. *Psychol. Methods* 18, 320–334. doi: 10.1037/a0032121
