# Supplementary material for: The initial development and validation of the Social Adaptability Skills Questionnaire: SASQ
Source: PLoS One. 2023 Aug 18;18(8):e0281971. doi: 10.1371/journal.pone.0281971 (PMC10437787; doi:10.1371/journal.pone.0281971)
Supplement: Attachment — Submitted filename: Rebuttal Letter Response to reviewers resubmission.docx [file pone.0281971.s007.docx]

**Rebuttal Letter (Response to reviewer)**

Thanks once again for your insights which have guided us to improve the manuscript.

| **Question** | **Response** |
| --- | --- |
| 1.  Did the authors reverse the negatively worded items’ values, this should be clearly stated | Yes, we did reverse the negatively worded questions in SPSS before analysis (i.e., 1 = 6, 2 = 5, 3 = 4, 5 = 2, and lastly 6 = 1). The following phrase has been added on the manuscript:  -*Prior to the analysis, scores of negatively worded questions were reversed*. |
| 2. Convergent validity (CV) analysis | Once again, thanks for raising the issue;  We have carried a CV analysis and additional paragraphs added on the manuscript.  Since we had to use the criterion values in reporting the CV. The literature reports no consensus as regards the best criterion. Several studies have reported different values with some reporting either “low” or “high” CV while criterion between *r* = .28 and *r* = .75 being accepted.  *Results*  The measures of family and club constructs (**0.761** and **0.732**) respectively reported high convergent validity. In contrast, both the measures of coach and teammates constructs (**0.631** and **0.657**) respectively reported average or modest convergent validity.  The following paragraph has been added to the manuscript to argue for the debatable CV criterion levels:  *Convergent validities above r = .70 are recommended, whereas those below r = .50 should be avoided (Carlson et al., 2012). However, research evidence suggests that actual levels of convergent validity in psychology research still vary widely (Carlson et al., 2012). Therefore, without more specific guidance, researchers reach logically inconsistent conclusions, arguing that a convergent validity as low as r = .28 (e.g., Larraza-Kintana, Wiseman-Meija & Welbourne, 2007) indicates the measures converge, whereas others report convergent analysis as high as r = .75 (e.g., Podsakoff, Whiting, Podsakoff & Baume, 2018) signalling high convergence*. |
| 3. Discriminant validity analysis not mentioned | -Thanks for the comment;  We have now performed a discriminant validity analysis using  the heterotrait-monotrait ratio of correlations (HTMT) as a new approach to assess discriminant validity in variance-based SEM (Henseler, Ringle & Sarstedt, 2015). This is due to the fact that recent research suggests that the routinely used Fornell-Larcker criterion is not effective under certain circumstances of discriminant validity testing (Henseler et al. 2014).  -HTMT procedure can be assessed through two ways (i) as a criterion, and (ii) a statistical test. We chose the latter due to the following reasons: (a) predefined criterion thresholds are debatable (Clark & Watson, 1995; Kline, 2016), and (b) statistical test involves bootstrapping which allows for constructing confidence intervals and also reducing familywise error rate.  *Results*  The computations yields values between (0.661 and 0.849) (Table 4). Upon comparing if the CI result value (one) falls outside the interval range, it can be reported that all the four constructs are empirically distinct. |
| Additional remarks | -First, it is important to note that the elimination of items purely on statistical grounds can have adverse consequences for the construct measures’ such as discriminant and convergence validity (e.g., Hair et al. 2014). Therefore, researchers should carefully scrutinize the scales (either based on prior research results, or on those from a pretest in case of the newly developed measures) and determine whether all the construct domain facets have been captured.  -Second, it is important to note, however, that discriminant validity is not exclusively an empirical means to validate a model.  Theoretical foundations and arguments should provide reasons for constructs correlating or not (Bollen and Lennox 1991). : a construct should rather be viewed as “something created from the empirical data which is intended to enable empirical testing of propositions regarding the concept” (Rigdon 2014, pp. 43–344). Consequently, any derivation of HTMT thresholds is subjective. On the other hand, concepts are partly defined by their relationships with other concepts within a nomological network, a system of law-like relationships discovered over time and which anchor each concept. Therefore, hindsight failure to establish discriminant validity between two constructs does not necessarily imply that the underlying concepts are identical, especially when follow-up research provides continued support for differing relationships with the antecedent and the resultant concepts (Bagozzi and Phillips 1982). Nevertheless, our research reported favourable discriminant validity results. |
